# Reviews and Book Notices

**Published:** 1880-09

**Authors:** 


### Reviews and Book Notices.

ARTICLE IX.—GUY'S HOSPITAL REPORTS. Edited by H. G. Howse, M.S., and Frederick Taylor, M.D. Third Series, vol. xxiv, 1879. J. & A. Churchill, London.

Guy's Hospital Reports need no introduction to the readers of any medical journal in the world. We have before us the 24th volume of the third series, a book of 496 pages, made up of twenty different articles, illustrated by nine plates and several wood cuts.

While we have neither time nor space for an exhaustive review of this most valuable "Report," we cannot lay it aside without calling attention to two papers which are alike remarkable for their originality and ability. We refer to "Art. XII, Constructive Inflammation and Ulcers; by C. H. Golding-Bird, B.A., M.B.," and "Art. XVII, Some of the Clinical Aspects of Chronic Bright's Disease; by F. A. Mahomed, M.D.

Article XII, by Golding-Bird, is richly worth a careful study by every student of pathology. It is an admirable exposition of what is at present known concerning the differentiation of protoplasm in "constructive inflammation," as it is seen in the repair of ulcers. Dr. Bird is evidently a biologist as well as a surgeon, and he handles his subject from the broad standpoint of one who is thoroughly acquainted with the laws of cell development. He regards, and very justly according to our way of thinking, "constructive inflammation," as essentially a "local living of embryonic life over again, a recommencement of evolution from a nucleated mass of protoplasm." And he immediately adds, "in this sense constructive inflammation or repair becomes a physiological rather than a pathological process." These two brief sentences indicate essentially what may be called the biology of the process of repair, excluding of course the process of "immediate union," which is so exceeedingly rare that many surgeons deny its existence, and many others confound it with "union by first intention."

"In tracing out briefly the steps that occur in repair," says Mr. Golding-Bird, "it will be shown how exactly in detail they are the repetitions of early development, and in this sense also physiological."

The relations existing between embryonic development and the cell-differentiations of repair, are stated as follows:

"The blastoderm cells in the ovum, or the earliest cells of the embryo—hence called the embryo cells—are simple nucleated protoplasmic spheres or cells, capable of amœboid movements (Embryonic tissue).

"Later on, by these movements they acquire branches, and these unite into a tissue (Granulation tissue).

"Still later, this last becomes differentiated into fibrous tissue (Cicatricial tissue).

"All these conditions may be traced in the fully formed animal, in the constructive inflammatory process; its very existence depends upon their fulfillment."

We have not seen the broad principle that reparative processes in the adult are but the essential equivalent of embryonic cell-development; that cell-growth is cell-growth everywhere and always; that all cell multiplication and cell differentiation in the adult and in the embryo, are essentially one; that constructive growth in the adult is but the repetition of constructive growth in the ovum, so clearly stated before. Nevertheless, we have *taught* this doctrine for nearly ten years, not only with respect to so-called "constructive inflammations," but also in in relation to other processes which are classed as "pathological." To a very much greater extent than our text books indicate are "pathology" and "physiology" identical; to a very much greater extent than appears on the surface are the processes of embryological growth repeated in the adult body, both "physiologically" and "pathologically." Hence we have for years past urged upon students the importance of understanding the history of development as it is seen in the ovum.

Mr. Golding-Bird has some peculiar notions respecting the pus-corpuscle, and they are worth examining. In order to make his meaning clear we are compelled to quote somewhat at length.

"Although," says the author, "the name 'pus-corpuscle' has been given as synonymous with embryo cell, yet as a pus cell it requires here, more from its clinical than its histological aspects, some further and special mention. Microscopically there is no distinction between the pus cell and those with which it has been classed. The embryo-cell contains one or more (usually four) nuclei, whether it be taken from the tissues or immediately from the blood, although it requires generally the addition of acetic acid to exhibit them. *The pus-corpuscle has always four nuclei, hence it is the same as saying that the pus-corpuscle is only a four-nucleated embryo-cell.*

"To the surgeon the pus cell is a cast off and dead element; and as we see it with the naked eye so it is, but in situations only penetrable by the microscope, *it is a living entity, and enjoying all the amœboid and locomotive characters of the one-nucleated cell.* (The italics are our own.) But why should the pus-cell contain four nuclei? Or why should pus—clinical pus that is—be composed solely of four-nucleated cells?

"Clinical observation here assists the histological, and if the answer to be given does not explain why a healthy white blood corpuscle may have four nuclei, it is yet amply sufficient for the subject under consideration. When a protoplasmic cell divides, the process commences in the nucleus, hence an embryo-cell (pus) that contains four nuclei may be regarded as one in which multiplication has been checked at the first stage. Through lack of nutrition there is not sufficient energy on the one hand, nor protoplasm on the other, to complete the process, though in all other respects the cell retains its vital functions. The general state of health, as well as local conditions, therefore, may be expected to influence the amount of pus, and this every surgeon knows to be the case; pus in excess meaning nutrition so perverted that a greater or less amount of protoplasm is no longer able to be fixed in the tissues, and is therefore thrown off and lost."

According to our observation, although the pus cell frequently contains four nuclei, it is by no means an invariable rule. Very many pus cells never get beyond being "nuclei," biologically considered, yet they are potentially "pus" cells. There is really no fixed and unalterable law governing the development of pus cells; hence there can be no invariable rule as to the number of their nuclei. Mr. Golding-Bird's explanation of the presence of four nuclei in pus cells seems to us exactly no explanation at all. In fact it utterly fails to explain what is perhaps the most common mode of the development of pus cells, in the early stage of suppuration, in acute parachymatous inflammations, namely, by budding or "gemmation."

The remainder of this interesting paper is devoted to the discussion of "granulation tissue and granulations," "epitheliation" and the "pathology of granulation tissue," and each one of these subjects is handled with marked ability. We cannot agree with the author that "epithelial cells are developed from the embryo cells of the granulation tissue," but rather that they are developed from the bioplasts of previously existing epithelial cells; that is, we are strongly inclined to believe that the "doctrine of epithelium from epithelium" is the true doctrine after all. While we notice nothing in either of these three concluding sections which merits especial commendation or invites criticism, we take pleasure in advising every student of pathology to read carefully and thoughtfully the entire essay upon "Constructive Inflammation and Ulcers," with the assurance that it will be found equally pleasant and profitable. I. N. D.

ARTICLE X.—BRAINWORK AND OVERWORK. By Dr. H. C. Wood, Clinical Professor of Nervous Diseases in the University of Pennsylvania. Philadelphia: P. Blakiston; 1880.

This book is worth reading carefully. In the second chapter of the work the author considers the causes of nervous diseases. "Bad food, bad water, habitual filth, and living in badly ventilated, damp or dark houses," are among the etiological forces behind many nervous and brain disorders.

Dr. Wood divides the causes of nervous diseases into two great classes, viz: exposure and dissipation. In considering the influence of hard work as a cause of nervous trouble, the author records it as his firm belief that this factor is greatly overestimated. There are a few pages (commencing with the fiftieth) of great interest, in which the author discusses the manner in which mental labor affects the brain. Chapter IV is devoted to the discussion of "Rest in Labor." Chapters V and VI to "Rest in Recreation and Sleep."

The book, written in a pleasing style, free from technicalities, is one which will interest the general reader and contains in its 125 pages a large amount of information upon several important themes.

There seems to be a desire, upon the part of the writer, to impress the fact on every reader of the book that hard work, in certain limits, is not only not hurtful but necessary to health; and that on the contrary, worry and waste of nervous and physical forces are deadly foes to health. The chapters are too short to give a satisfactory discussion of their several titles, but the intelligent reader can glean much useful information from the book.

B. W. G.

ARTICLE XI.—OBSERVATIONS ON FATTY HEART. By Henry Kennedy, A.B., M.B., Univ. Dublin; Fellow of the King and Queen's College of Physicians in Ireland; Physician to Simpson's Hospital, etc. Dublin: Fannning & Co.; 1880.

This is a small volume of 171 pages, treating of the morbid etiology, anatomy, symptoms and diagnosis, prognosis and treatment of fatty heart.

The author recognizes two varieties of this affection, both of which he believes dependent on a fatty diathesis and the manifestations of a general disease rather than a local disorder.

The subject is quite thoroughly considered, and whatever clinicians may think of the author's propositions, they are certainly of great interest and merit careful consideration.

In the diagnosis of fatty deposit on the heart the main reliance is placed on: 1st, a slow, full pulse; 2nd, enlargement of the area of cardiac dullness; 3d, the fact of the individual being fleshy; and 4th, a soft soufflet with the first sound of the heart in the aortic area whenever the valves are affected.

In the diagnosis of the fatty degeneration of the muscular fibers of the organ he relies mainly on: 1st, a slow pulse, often less than 50 per minute; 2d, weak impulse of the heart apex; and 3d, weakness of the heart sounds, the organ meanwhile retaining its normal size.

The only other disease causing these two latter signs are pericarditis and dilatation of the heart, but in both these there is an increased area of dullness and a more or less rapid pulse. In rare instances the heart-sounds in fatty degeneration are distinct and rapid, but they then have a metallic quality and seem distant like the fœtal heart.

The nervous and other symptoms attending these two varieties are much alike and are always of importance in forming a diagnosis. The principal of these are dispnœa, loss of strength, flabby condition of the muscles, uneasiness or pain in the region of the heart or diaphragm, hesitating or slow speech, and not infrequently pseudo-apoplexy, in which the patient, without any warning, falls as though shot and presents, in more or less perfection, the ordinary symptoms of apoplexy, the characteristic feature being that the loss of consciousness or paralysis are only temporary; at first usually disappearing in a few moments, and later after, at most, an interval of a few days.

The book is well worth the careful perusal of every practitioner as it deals with a subject too litte understood and especially with a diagnosis which is not usually made until the autopsy. E. F. I.

ARTICLE XII.—OUR HOMES. By Henry Hartshorne, A.M., M.D., Formerly Professor of Hygiene in the University of Pensylvania, etc., etc. Philadelphia: Presley Blakiston, 1012 Walnut St.; 1880.

This book is one of the series of American Health Primers published by the enterprising Philadelphia house whose name appears above. The author of the book is an eminent authority upon the subject about which he writes.

Dr. Hartshorne has given to the public, in a concise, condensed and practical form, the results of his thought (as well as that of others) upon a very important topic. It is no less important a theme than that of the situation, construction, ventilation, lighting, warming and drainage of dwelling houses.

Preventive medicine must, as the masses become more intelligent, become an important factor in medical literature. It is of great consequence to know how to prevent disease. There is no doubt that a large percentage of disease and death grows out of utter ignorance in regard to the laws of health. This being so, hygiene is an all-important department of medicine. The writer has made no pretense of producing an elaborate and exhaustive treatise on the subjects which he introduces. He has, the rather, condensed and sifted the literature which has been written upon these topics; and, as a result, has made a book, which, while it is instructive and interesting, is not dull nor burdened with unimportant facts. The book is a résumé of the writings of English, German, and American authors on Hygiene. It touches only the salient points of the subject which it discusses.

The chapter on the situation of dwellings contains some suggestive remarks. The dangers from malaria, carbonic acid gas, surface defilement, etc., are strongly set forth. The faulty construction of houses is asserted to be, in many instances, a fruitful cause of disease.

The author quotes experiments from the eminent Bavarian experimenter, Prof. Pettenkofer, which show how ground air is brought into dwelling houses and becomes an invisible and unsuspected foe to health and comfort. The use of wall papers containing arsenic is condemned in vigorous terms.

The subject of drainage, to which the writer devotes the longest chapter in the book, is presented more fully and elaborately than any of the other topics, which, taken together, form the book. This chapter contains quite a detailed account of different systems of plumbing and draining, and the reader can glean from it, by a careful perusal, all the really important points relating to the subject of drainage. There are, of course, other and more elaborate works, which treat this subject exhaustively. In short, this book is suggestive rather than exhaustive, brief and to the point, fully up to the times, and will repay a careful reading.

